# The Application of Internet of Things Data Analysis in the Development of International Trade

**DOI:** 10.1155/2022/5507951

**Published:** 2022-06-20

**Authors:** Hao Qiuxia, Hou Yujie

**Affiliations:** School of International Business, Qingdao Huanghai University, Qingdao 7266000, Shandong, China

## Abstract

There are some problems in the application of current data analysis methods in international economy and trade, such as low service efficiency, low data utilization, and low degree of intelligence. Based on this, this paper studies the application of the Internet of things data analysis method in international trade development and economic and industrial growth. Firstly, the Internet of things economic data analysis model (IOT-EET model) based on simulated annealing early warning algorithm is established to store and analyze the data in the whole chain of international trade. Then, combined with the analysis methods of international trade economic data over the years, it is fed back to the IOT-EET model for error calibration. Finally, relevant experiments are designed to analyze the correlation between international trade development and national economic growth. The results show that compared with the traditional method based on module data analysis, this IOT-EET model can realize the correlation matching analysis of the data involved in the development of international trade in combination with the Internet of things technology and analyze the factors affecting international trade transactions. Therefore, it has the advantages of good reliability and strong pertinence.

## 1. Introduction

At present, under the impact of the new generation of information and communication technology, the Internet of things is accelerating its penetration into urban management, new energy, medical treatment, manufacturing, and other fields. Especially in recent years, with the emergence of developed countries and regions in Taiwan, the relevant policies of the Internet of things have been strategically laid out. We hope to seize the opportunity in the new round of information industry development, and the Internet of things industry in Tongqiu is growing rapidly. The Internet of things has become the third wave of the world information industry after computers and the Internet. With the advent of economic globalization and the rapid development of Internet of things data analysis technology, international trade also presents great changes in data analysis methods and business processing [1]. In recent years, with the rapid development of Internet of things technology and the emergence and application of trade economic analysis methods and the IOT-EET model, international trade is also facing an important transformation of digital development [2]. Therefore, how to share trade data with high accuracy has become an important indicator to measure the accuracy of trade data [3]. On the other hand, although there are many international trade economic growth analysis models, they can not completely solve the practical problems [4]. For example, in the analysis of the development potential of international trade, most of the current analysis models belong to customized analysis models, so there will be large loopholes in the actual application process [5]. Based on this background, this paper studies the interaction degree analysis system of international trade and economic development and puts forward the Internet of things economic data analysis model (IOT-EET model) based on Internet of things data analysis, which can quantitatively analyze and characterize the internal interaction degree of international trade and data analysis.

In this paper, the application of the IOT-1 algorithm in the process of international economic growth is divided into four parts: slow economic growth and low efficiency of international trade.  Section 1 introduces the research background, research route, and innovation.  Section 2 objectively introduces the evaluation methods of international trade economic growth and the research status of data mining methods in international trade. In Section 3, the IOT-EET model based on simulated annealing early warning algorithm is constructed, and the high-dimensional multivariate analysis system is established by using the “Newton avige” high-dimensional equation method.  Section 4 tests the data peacekeeping system of international trade and economic growth constructed in this paper, analyzes the results, and draws a conclusion.

At present, the mainstream international trade economic growth analysis model (mainly a quantitative data analysis model based on an intelligent dimension collaborative algorithm) has the disadvantages of small application scope and uncontrollable error rate. The innovation of this paper is to build a data analysis model of the correlation between international trade and economic growth through simulated annealing early warning algorithm, combined with the IOT-EET model in the field of artificial intelligence big data and Internet of things technology. On this basis, the model can not only record and store the data between international trade and economic growth in multiple regions, but we can also make full use of the characteristics of economic growth between each region and the normalization standard of existing economic models to realize intelligent analysis through the IOT-EET model. On the other hand, using Newton factor quantitative index to complete the factor reliability analysis of international trade and economic growth can effectively reduce the error analysis of trade and economic data.

This paper studies the application of the Internet of things data analysis method in the development of international trade and economic and industrial growth. The economic data analysis model of the Internet of things (IOT-EET model) based on simulated annealing early warning algorithm is established. The innovation contribution is that the model has the advantages of high precision and wide application range. Compared with the traditional research methods of international trade and economic growth based on regional unconventional data analysis, the IOT-EET model can realize high-dimensional reliability analysis and adjustment in the process of the impact of international trade on the local economy. It is helpful for the application of current data analysis methods in international economy and trade. It provides a solution reference for the problems of low service efficiency, low data utilization, and low degree of intelligence.

## 2. Related Work

In the process of market-oriented economic analysis of existing international trade, there are problems of low data utilization, slow economic growth, and low service efficiency. Most scholars have innovated the internal data analysis methods of international trade and economic growth and tried to solve the above problems [6]. According to the characteristics of the existing international trade system and the differentiated characteristics of economic globalization, Rocki et al. build a data analysis model based on the method of national system construction to realize the analysis and quantitative representation of the responsibilities of different countries in international trade [7]. Aiming at the low efficiency of data analysis strategy in the process of international trade and economic development, according to the macrocontrol strategy in economics, Rocki et al. put forward an international trade and economic analysis dam model based on high-latitude analysis strategy, which realizes the high accuracy analysis of trade data by combining the idea of the marginal effect of dam burst [7]. Lin et al. found that most countries have large trade deficits in the process of international trade transactions, so they proposed an international trade rationalization system based on national conditions and macroeconomic theory. The system clearly combines the Internet of things data analysis technology to realize the rationalization prediction of international trade, but it needs to know the economic data of different industries [8]. Cao and other scholars put forward an Internet of things data analysis model combining artificial intelligence technology and strategy in order to realize the high-latitude application and analysis of data analysis methods in the trade economy. The model can predict the economic demand of different industries through trade data information, but the accuracy of prediction is related to the type of input parameters [9]. In order to effectively improve the accuracy of international trade, Peng et al. realize the interconnection of economics and trade in different regions through the Internet of things technology and then realize the calibration and control of different types of databases with the help of data analysis strategy [10]. Aiming at the problem of low data utilization in the development of international trade, Hoffmann C and other scholars put forward an orthogonal decomposition matching adaptive model, which can effectively insert and guide different types of databases and realize high accuracy analysis of different trade data information [11]. Siddig and other scholars achieve high accuracy at different levels through serial interworking of various types of data [12]. In order to further study the influencing factors in the process of international trade and economic development, scholars such as Gilliland combined with the idea of the economic data analysis model of the Internet of things proposed a serial interworking method based on reliability analysis. This method can effectively improve the potential income space of international trade, but the industry needs to be limited [13]. Aiming at the problem of high cost growth of international trade, Rui Wa and other scholars adopted different types of correlation data models and proposed an ultrahigh-precision data exchange method. This method can effectively improve the high-precision data characteristics of different trade data, but it is necessary to conduct high-intensity analysis on different data analysis [14]. According to the averaging shortcomings of different international trade in development, Paroussos and other scholars put forward a collaborative innovation method of high-value trade based on the CNN network. Experiments show that international trade can indirectly support the improvement of the local economy [15]. In order to solve the problems of the high-value analysis industry in the development of international trade, Feuerbacher A and other scholars put forward an economic growth innovation evaluation model based on the characteristics of regional development, so as to strengthen the analysis and intelligent management of the construction of international trade system [16].

Based on the above research results, we can know that the current mainstream international trade economic growth analysis model (mainly based on quantitative data evaluation and analysis model) has the disadvantage of the small scope of application [17–19]. On the other hand, in the quantitative evaluation of international trade and economic development, different types of innovative analysis methods have high-value cultivation strategies with low value and low accuracy, which leads to the improvement of the overall computational complexity and the internal analysis efficiency [20, 21]. Therefore, it is of great significance to study the application of the IOT-EET model based on the Internet of things data analysis strategy in the development of international trade.

## 3. Methodology

### 3.1. Application of IOT-EET Model Based on Simulated Annealing Early Warning Algorithm in Economic Growth Analysis

**Figure 1:**
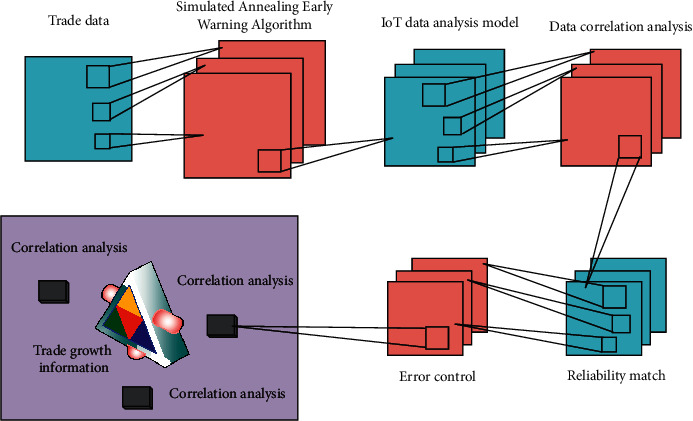
Operational analysis principle of IOT-EET model based on simulated annealing early warning algorithm.

Simulated annealing early warning algorithm is a commonly used algorithm for data analysis. This method solves the calculation problems with high dimension and large amount of data by simulating the idea of how to effectively extinguish the fire source in the effective space and realizes the sampling solution of complex problems. The Internet of things economic data analysis model (IOT-EET) includes three significant features. The first is “interactivity,” which guarantees and stipulates the iterative data acquisition method of economic entities. In this model, one of the representative features is to maximize the use of utility economic data. International trade also follows the decision-making principle of cost minimization and also includes economic entities such as government, trade organizations, importers, and exporters, which respond to price changes [22]. Secondly, it is “discrete,” which means that it includes the discrete analysis of demand and supply. Many prices in the model are determined by both supply and demand, and the price change finally makes the market realize equilibrium [23]. Finally, it is “normalized” because the model reflects the actual trade data and the economic problems existing in the actual region, which is closer to the current situation of economic development. In addition, it also involves industrial policy, income distribution, trade policy, etc. [24]. Therefore, the IOT-EET model combined with simulated annealing early warning algorithm can well solve the problem of super precision noncorrelation between international trade and regional economic growth. The data correlation analysis process of the IOT-EET model based on simulated annealing early warning algorithm in international trade and economic growth is shown in Figure 1.

### 3.2. Data Analysis Process of IOT-EET Model in International Trade and Economic Growth

In the process of international trade, the economic data of different regions have different characteristics, and their correlation also has certain laws. For example, the economic data of the logistics industry in regions with large exports to the manufacturing industry will increase more significantly. Therefore, after the IOT-EET model is established. It is necessary to quantitatively discuss the economic data analysis process of the IOT-EET model in international trade and economic growth.

According to the necessary conditions of the macroeconomic model in trade economics, it is necessary to design the upper limit value, coordination strategy, and error analysis system function of the IOT-EET model. Therefore, the upper limit function *R*(*x*) of economic growth, the synergy strategy function *T*(*x*), and the error analysis system function *Y*(*x*) are set as follows:

(1) $$\begin{array}{c} R\left(x\right)=\frac{\sum_{k=1}^{p}x_{k}^{2}/x_{k+1}^{2}/k{\bar{x}}_{p}+1/\sum_{p=1}^{k}k{\bar{x}}_{p}}{2},\\ T\left(x\right)=\frac{p!/1+{\bar{x}}_{p}+\sqrt{x_{1}+x_{k}+x_{p}/{\bar{x}}_{p+k-1}}}{{\bar{x}}_{p+k}},\\ Y\left(x\right)=\sum_{k=1}^{p}{\left(x_{k}-{\bar{x}}_{p}\right)}^{2}+\sum_{k=1}^{p}{\left(x_{k}+{\bar{x}}_{p}\right)}^{2}+\frac{1}{\left(k+1\right){\bar{x}}_{p}}, \end{array}$$

where *x*_*k*_ is the iterative international trade volume data, *p* is the overall number of international trade transactions, *k* is the number of international trade service types, and ${\bar{x}}_{p}$ is the average error data of regional quarterly trade and economic growth. After the standard characteristic analysis of the above system function, the corresponding expression is

(2) $$\begin{array}{c} R{}^{\prime }\left(x\right)=\frac{k-\sum_{k=1}^{p}x_{k}^{2}/x_{k+1}^{2}}{1+k{\sum_{k=1}^{p}\left(x+{\bar{x}}_{p}/1+{\bar{x}}_{p}\right)}^{2}},\\ T{}^{\prime }\left(x\right)=\sqrt{\frac{\left(p+1\right)!}{p+\bar{x}}+\frac{x_{1}+x_{k}+x_{p}}{{\bar{x}}_{p+k-1}}},\\ Y{}^{\prime }\left(x\right)=\sqrt{2+\sum_{k=1}^{p}{\left(x_{k}+{\bar{x}}_{p}\right)}^{2}+\frac{1}{1+k{\sum_{k=1}^{p}\left(x+{\bar{x}}_{p}/1+{\bar{x}}_{p}\right)}^{2}}}. \end{array}$$

**Figure 2:**
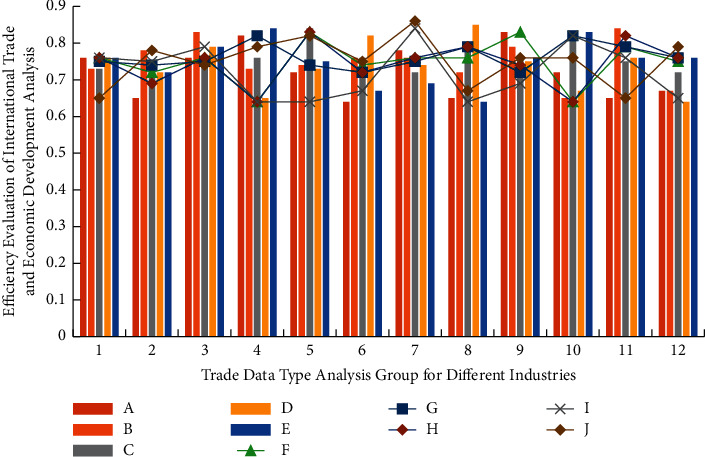
Simulation analysis results of the IOT-EET model on the analysis efficiency of international trade and economic development.

After completing the regional international trade indicators, it is necessary to retrieve the correlation matching characteristics of different types of international trade and economic growth data and realize multidimensional division according to the growth rate. After ultrahigh analysis, it is necessary to perturb and match its internal trade deficit data and input its data center group into the IOT-EET model. The simulation analysis results of the simulation data group on the analysis efficiency of international trade and economic development are shown in Figure 2 (where *A*-*J* is the standard discriminant index with 10 groups of dimensions gradually increasing).

It can be seen from Figure 2 that among the 12 groups of databases, the internal relevance is quite different from the dimension of 10 indicators, but the internal data change trends are similar, both within a relatively stable change range, and the data consistency of the fourth group of databases is the highest, because after setting the operation threshold. It is also necessary to give priority to the relevant data affecting international trade and economic growth in combination with different types of matching degrees and combine them with numerical change indicators for variable weight analysis. The trade deficit tracking function *F*(*x*), economic growth classification function *G*(*x*), and trade error evaluation function *H*(*x*) used in the vector comparison process in the above simulation analysis stage are

(3) $$\begin{array}{c} F\left(x\right)=\sqrt{1+\frac{\sqrt{{\left|x\right|}^{2}+m\left|x\right|+\sqrt{\left|x\right|}}}{\sqrt{k\left|x\right|}+m{\left|x\right|}^{2}+\sqrt{k}}},\\ G\left(x\right)=\frac{\sqrt{m{\left|x\right|}^{2}+m\left|x\right|+\sqrt{\left|x\right|}}+\sqrt{\sum_{k=1}^{m}1/m\left|x\right|+k{\left|x\right|}^{2}+\text{mk}}}{3\text{mk}},\\ H\left(x\right)=\frac{\sqrt{kG^{2}\left(x\right)+mF\left(x\right)/kG\left(x\right)+\text{mx}}+\sqrt{\left(1+H\left(x\right)/\text{km}\right)}}{m}, \end{array}$$

where |*x*| is the high-precision matching grade index of international trade economic data vector, *m* and *k* are the type and upper limit value of data in the process of international trade economic growth, respectively. After fuzzy analysis and processing of the above-mentioned three functions, the corresponding expression is

(4) $$\begin{array}{c} F{}^{\prime }\left(x\right)=\frac{{\left|x\right|}^{2}+m\left|x\right|+\sqrt{\left|x\right|}}{k\left|x\right|+m{\left|x\right|}^{2}+k},\\ G{}^{\prime }\left(x\right)=k\sqrt{\sum_{k=1}^{m}\frac{\sqrt{m{\left|x\right|}^{2}+m\left|x\right|+\sqrt{\left|x\right|}}}{m\left|x\right|+k{\left|x\right|}^{2}+\text{mk}}}+\frac{1}{\sqrt{m\left|x\right|}+k{\left|x\right|}^{2}+\text{mk}},\\ H{}^{\prime }\left(x\right)=\frac{G\left(\text{kx}\right)}{mF\left(\text{kx}\right)+kH\left(\text{kx}\right)}+\sum_{k=1}^{m}\frac{H{}^{\prime }\left(x\right)}{mF\left(\text{kx}\right)+kG\left(\text{kx}\right)}. \end{array}$$

**Figure 3:**
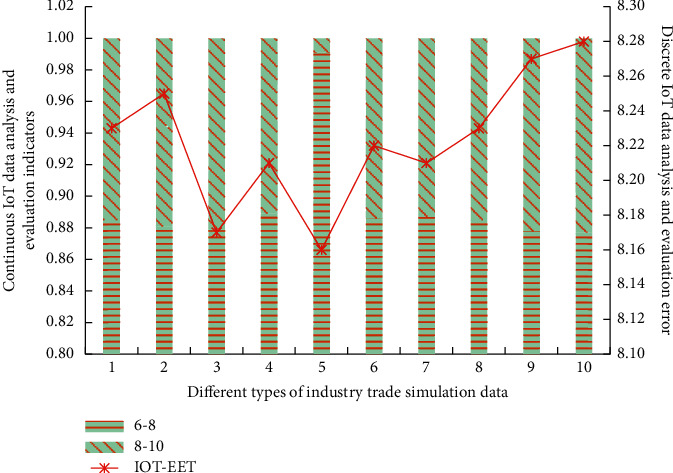
Economic growth simulation analysis of two types of random international trade economic data by IOT-EET model.

In this stage, the simulation analysis results of the IOT-EET model on the economic growth of two types of random international trade economic data are shown in Figure 3.

It can be seen from Figure 3 that under the IOT-EET model based on simulated annealing early warning algorithm, there are certain differences in international trade and economic data among different regions. Specifically, the content is inconsistent with the value of international trade and economic information stored in the cloud in terms of analysis type and standardized evaluation.

The existing computer cannot reach the level of complete ultrahigh precision in the process of matching and analyzing trade data. With the increase of simulation analysis times, the internal correlation evaluation and error analysis of different types of data groups show different trends. Different types of intersections appear under different simulation times.

### 3.3. Simulation Verification Process of International Trade Economic Growth Analysis Model Combined with IOT-EET Model

**Figure 4:**
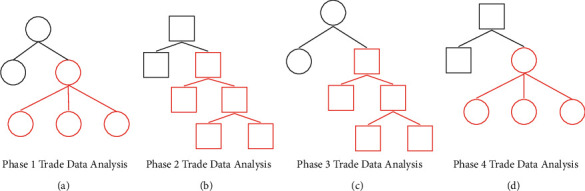
The process of analyzing the data package of multiple economic differentiation characteristics within international trade. (a) Phase 1 Trade data analysis. (b) Phase 1 Trade data analysis. (c) Phase 1 Trade data analysis. (d) Phase 1 Trade data analysis.

After adopting the trade economic data analysis model based on Internet of things technology and simulated annealing early warning algorithm, in order to further analyze the internal relationship between regional trade economy and industry development, it is also necessary to reverse verify the analysis results of its international trade differentiated data in combination with the Internet of things data verification strategy, Therefore, it is necessary to analyze a number of economic differentiation characteristic data packets within international trade. The analysis process is shown in Figure 4.

**Figure 5:**
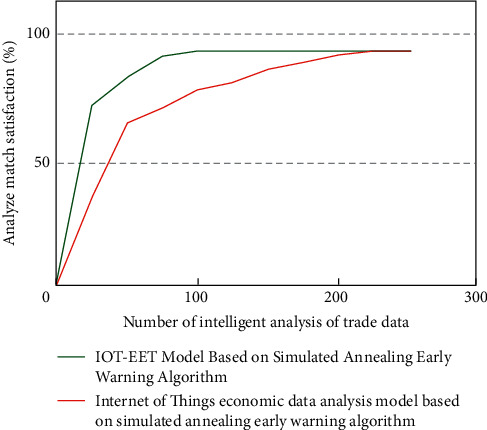
Classification simulation analysis results after correction of two different models.

In the international trade and economic transaction data of different industries (such as manufacturing industry, household industry, and digital industry), the corresponding identification error rate and correlation evaluation results are different, because the calculation and identification of different types of similarity can be based on the difference and similarity of international trade data information, and intelligent high-precision classification is carried out through the economic data analysis model of the Internet of things. The classification simulation analysis results after correction are shown in Figure 5.

It can be seen from Figures 4 and 5 that the classification effect and coupling correlation degree of the data analysis results of local and regional international trade data under the IOT-EET model are different because the data obtained by the IOT-EET model analysis method have carried out the high-precision standardized classification of different types of data groups in the process of analysis. Therefore, when it is reflected in the final data analysis results, the corresponding differences will not change greatly.

Finally, in the prediction and analysis of international trade volume data, the trade accuracy evaluation function *C*(*x*) and noncorrelation function *V*(*x*) based on simulated annealing early warning algorithm are introduced, and their mathematical expressions are as follows:

(5) $$\begin{array}{c} C\left(x\right)=\sqrt{\frac{1}{\sqrt{\sum_{j=1}^{p}\left(jx_{j}^{2}+{\bar{x}}^{3}\right)}}+\frac{1}{\sqrt{\sum_{k=1}^{p}\left(kx_{k}^{2}-{\bar{x}}^{2}\right)}}},\\ V\left(x\right)=\sum_{k=1}^{p}{\left(kx_{k}^{2}-{\bar{x}}^{2}\right)}^{2}-\frac{1}{1+p\bar{x}}, \end{array}$$

where *x* represents the cluster samples of different trade and economic growth data groups, *k* and *j* represent different data cluster numbers, and *p* represents the maximum number. In the process of reducing the error of the IOT-EET model and correcting the results of the overall international trade operation and analysis function, it is necessary to normalize the sample data. The intelligent data set of trade center classification used in this process is shown in the following expression:

(6) $$\begin{array}{c} P\left(x\right)=\frac{l+\text{rx}}{lx+r}+\frac{\sum_{i=1}^{m}\left(x-1\right)+\sum_{i=1}^{r}x}{m+r}. \end{array}$$

Here, *x* represents different types of international trade and economic growth data sets, *l* and *r* represent different trade transaction rule factors.

## 4. Result Analysis and Discussion

### 4.1. Confirmatory Test of Correlation Analysis between International Trade and Economic Growth Based on IOT-EET Model

**Figure 6:**
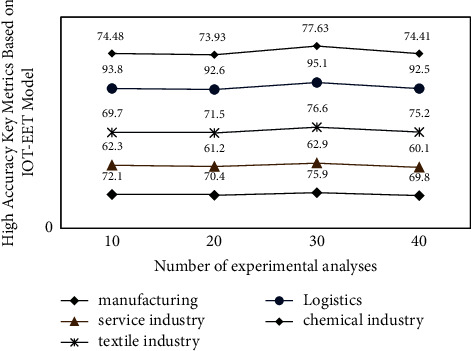
Preliminary experimental analysis results of 3 sets of experimental data.

After constructing the international trade and economic growth evaluation system based on the IOT-EET model, in order to further verify the accuracy and error of its analysis model, this study sets different parameters to verify the data, and the internal correlation between different data groups is random. Therefore, for different types of trade data indicators, their internal relevance shows an obvious change trend. The preliminary analysis results of international trade data of different industries during the experiment are shown in Figure 6, in which the horizontal axis represents the number of experiments and the vertical axis represents the key measurement indicators of high accuracy based on the IOT-EET model.

It can be seen from Figure 6 that under the simulated annealing early warning algorithm, with the increase of the number of experiments, the analysis type data of international trade show different types of change trends, and the number of internal correlation consistent data shows a trend of decreasing first and then stable, compared with the data group without correction factor. There are more standardized correlation degree and economic growth data in the international trade data set with a correction factor, because the operation method adopted this time is the international trade economic correlation analysis model of the improved IOT-EET model combined with the simulated annealing early warning algorithm, and the threshold parameters of different numbers of data envelopment sets are set. To characterize the relevance and relevance between international trade and local economic growth.

### 4.2. Experimental Results and Analysis

**Figure 7:**
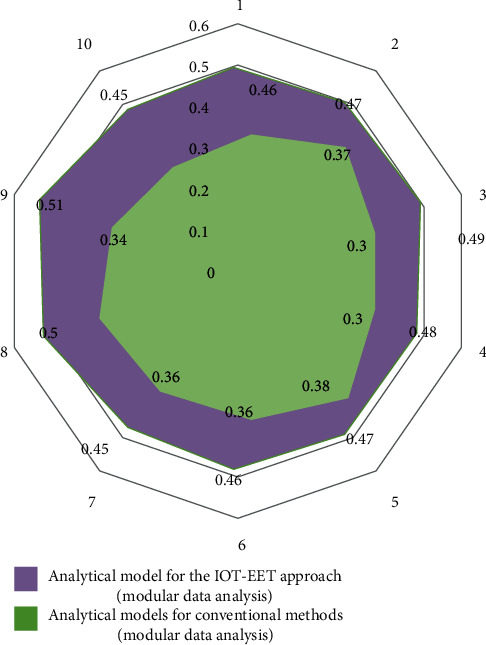
Analysis of the correlation error degree of the experimental results.

In the process of analyzing the experimental results, this study is based on the revenue and expenditure data disclosed by all scenic spots in Beijing in recent 5 years. The experimental group adopts the IOT-EET model and the control group adopts the analysis model of the conventional method (modular data analysis). The analysis results are shown in Figure 7. In Figure 7, purple represents the analysis model of the IOT-EET method, and green represents the analysis model of the conventional method.

Through the analysis of the results in Figure 7, it can be found that under the IOT-EET model, in the process of efficient exchange of different types of international trade data, the error rate of the final result will be significantly reduced, which is due to the significant differences in the internal relevance of different types of data groups.

Compared with the traditional research methods of international trade and economic growth based on regional unconventional data analysis, the EET model of the Internet of things can realize high-dimensional reliability analysis and adjustment in the process of the impact of international trade on the local economy. This is helpful to the application of current data analysis methods in international economy and trade. It provides a reference for solving the problems of low service efficiency, low data utilization, and low degree of intelligence.

## 5. Conclusion

At present, there are some problems in international trade in terms of service volume and economic growth, such as low service efficiency, poor coordinated development, and slow economic growth. Compared with the problems of low data analysis dimension and poor reliability in the current mainstream international trade economic growth analysis model, this paper studies the correlation analysis system between international trade and economic industrial growth based on Computable General Equilibrium (IOT-EET) model. Firstly, the IOT-EET data analysis model based on simulated annealing early warning algorithm is established to carry out multiscale collaborative processing of different types of international trade data information to realize the intelligent classification of the data. Then, combined with the international trade economic data and local economic growth rate in previous years, it is fed back to the IOT-EET model for error analysis, Finally, relevant experiments are designed to analyze the matching and reliability of international trade and local economic growth. The results show that compared with the traditional research method of international trade and economic growth based on regional unconventional data analysis, this IOT-EET model can realize high-latitude reliability analysis and adjustment in the process of the impact of international trade on the local economy. Therefore, it has the advantages of high accuracy and wide application range. However, this study does not consider the multiple security of international trade data. Therefore, in terms of strengthening the security of international trade and regional economic growth in different regions, an in-depth dimensional analysis is needed.

## Data Availability

The data used to support the findings of this study are available from the corresponding author upon request.

## References

[1] Tziavos N. I., Hemida H., Metje N., Baniotopoulos C. (2019). Non-linear finite element analysis of grouted connections for offshore monopile wind turbines. *Ocean Engineering*.

[2] Evangelos T., Antonakis A. N., Ismini B. (2021). ProteoSign v2: a faster and evolved user-friendly online tool for statistical analyses of differential proteomics. *Nucleic Acids Research*.

[3] Khalid F., Hasan S. R., Zia S., Hasan O., Awwad F., Shafique M. (2020). MacLeR: machine learning-based runtime hardware trojan detection in resource-constrained IoT edge devices. *IEEE Transactions on Computer-Aided Design of Integrated Circuits and Systems*.

[4] Staszewski R. B., Liu Y. H., Purushothaman V. K., Bachmann C. (2019). Design and analysis of a DCO-based phase-tracking RF receiver for IoT applications. *IEEE Journal of Solid-State Circuits*.

[5] Zhang T., Ma Y., Li A. (2021). Scenario analysis and assessment of China’s nuclear power policy based on the Paris Agreement: a dynamic CGE model. *Energy*.

[6] Li G., Zhang R., Masui T. (2021). CGE modeling with disaggregated pollution treatment sectors for assessing China’s environmental tax policies. *The Science of the Total Environment*.

[7] Pintor J. M. P., Garrinhas J. P. C. (2021). Eurocity Elvas, Badajoz and Campo Maior (EUROBEC): the socio-economic and territorial reality of a new cross-border governance structure in Portugal. *Journal of Interfaces in Arts and Culture*.

[8] Komarova V., Selivanova-Fyodorova N., Ruza O., Kaźmierczyk J. (2019). Modern trends in the economic differences between countries and within them: comparison of the world and the European Union. *Entrepreneurship and Sustainability Issues*.

[9] Cao Z., Liu G., Zhong S., Dai H., Pauliuk S. (2019). Integrating dynamic material flow analysis and computable general equilibrium models for both mass and monetary balances in prospective modeling: a case for the Chinese building sector. *Environmental Science & Technology*.

[10] Peng D., Yang Q., Yang H. J., Liu H., Zhu Y., Mu Y. (2020). Analysis on the relationship between fisheries economic growth and marine environmental pollution in China’s coastal regions. *The Science of the Total Environment*.

[11] Hoffmann C. (2019). Estimating the benefits of adaptation to extreme climate events, focusing on nonmarket damages[J]. *Ecological Economics*.

[12] Siddig K., Stepanyan D., Wiebelt M., Grethe H., Zhu T. (2020). Climate change and agriculture in the Sudan: impact pathways beyond changes in mean rainfall and temperature. *Ecological Economics*.

[13] Gilliland T. E., Sanchirico J. N., Taylor J. E. (2020). Market-driven bioeconomic general equilibrium impacts of tourism on resource-dependent local economies: a case from the western Philippines. *Journal of Environmental Management*.

[14] Rui W. A., Hd B., Yong G., Yang X., Xu T. (2019). Impacts of export restructuring on national economy and CO 2 emissions: a general equilibrium analysis for China. *Applied Energy*.

[15] Paroussos L., Fragkiadakis K., Fragkos P. (2020). Macro-economic analysis of green growth policies: the role of finance and technical progress in Italian green growth. *Climatic Change*.

[16] Feuerbacher A., Mcdonald S., Dukpa C., Grethe H. (2020). Seasonal rural labor markets and their relevance to policy analyses in developing countries. *Food Policy*.

[17] Liu H., Shladover S. E., Lu X. Y., Kan X. D. (2021). Freeway vehicle fuel efficiency improvement via cooperative adaptive cruise control. *Journal of Intelligent Transportation Systems*.

[18] Karavaeva I. V., Ivanov E. A., Lev M. Y. (2021). Modern trends for assessing the maximum permissible indicators of economic security in Russia. *Economics and Management: Problems, Solutions*.

[19] Dogru T., Marchio E. A., Bulut U., Suess C. (2019). Climate change: v. *Tourism Management*.

[20] Ramos V., Ruiz-Pérez M., Alorda B. (2021). A proposal for assessing digital economy spatial readiness at tourism destinations. *Sustainability*.

[21] Matyushok V., Krasavina V., Berezin A., Sendra García J. (2021). The global economy in technological transformation conditions: a review of modern trends. *Economic Research-Ekonomska Istraživanja*.

[22] Li X., Celotto S., Pizzol D. (2021). Metformin and health outcomes: An umbrella review of systematic reviews with meta-analyses. *European Journal of Clinical Investigation*.

[23] Hu H., Tang L. (2020). Edge intelligence for real-time data analytics in an IoT-based smart metering system. *IEEE Network*.

[24] Chae B. K. (2019). The evolution of the Internet of Things (IoT): a computational text analysis. *Telecommunications Policy*.

